# Everyday Discrimination and Vulnerability to HIV Transmission Among Sexual and Gender Minorities of Color in Raleigh-Durham, North Carolina

**DOI:** 10.1007/s10461-025-04839-z

**Published:** 2025-08-14

**Authors:** Dalton M. Craven, Ann M. Dennis, Justin Quimbo, Kham S.K. Piang, Britt Skaathun

**Affiliations:** 1 Division of Infectious Diseases, University of North Carolina at Chapel Hill, Chapel Hill, NC USA; 2 Division of Infectious Diseases and Global Public Health, Department of Medicine, University of California, San Diego, La Jolla, CA USA

**Keywords:** Discrimination, LGBTQ health, Race minority, HIV vulnerability, Prevention

## Abstract

Sexual and gender minority (SGM) individuals of color face disproportionate HIV burdens in the United States, partly due to the effects of discrimination. Discrimination may drive behaviors linked to HIV risk, including increased sexual activity and substance use, but these relationships remain underexplored in the Southern U.S. We conducted a cross-sectional analysis using data from a social network survey of adult persons of color assigned male sex at birth (AMAB) who have sex with other AMAB individuals in Raleigh-Durham, North Carolina. Everyday discrimination was measured using the five-item Everyday Discrimination Scale (EDS), summed by the number of situations reported. Participants were categorized by sexual activity level—high (≥ 3 partners in the past 6 months) or low (0–2 partners). Multivariable logistic regression was used to assess associations between EDS scores, substance use, and sexual activity. Among 100 participants (median age 32), 79% identified as Black/African American, 22% as Latinx, 55% were living with HIV, and 10% identified as gender diverse. Most (87%) reported experiencing at least one type of everyday discrimination in the past year. EDS scores were significantly higher among those with high sexual activity (median 4 vs. 3, *p* = 0.007). In adjusted models, both EDS (OR 1.76; 95% CI 1.25–2.61) and recreational drug use (OR 4.69; 95% CI 1.59–15.5) were associated with high sexual activity. Discrimination and substance use are significantly associated with elevated sexual activity among SGM of color. Multilevel interventions addressing these factors are needed to improve HIV prevention outcomes in this population.

## Introduction

In the Southern United States, HIV incidence is disproportionately high with about 49% of new HIV infections coming from the region, despite only comprising about 38% of the United States’ population [1, 2]. While the increased prevalence of HIV in the South is not fully understood, restricted expansion of Medicaid, health-care provider shortages, low health literacy, and HIV stigma may be contributory factors [3]. Further, profound disparities exist both geographically and demographically as sexual and gender minority (SGM) populations, especially those in racial and ethnic minority groups, experience an increased burden of HIV [4]. Over the past decade, the literature has shown that differences in rates of HIV among Black versus White SGM may be explained by differences in sexually transmitted infections (STI)s, undiagnosed HIV, and access to and retention in HIV prevention and treatment services [5]. While the reason is unclear, Black SGM have a higher prevalence of STIs, which may increase ones risk of HIV acquisition. Furthermore, a lack of HIV testing or consistent HIV prevention programming in SGM populations may perpetuate the increasing burden of HIV. However, the impact of discrimination on HIV vulnerability (acquisition or transmission risk) in SGM is not well understood and warrants further investigation and is the objective of this analysis.

Stigma, discrimination, and minority stress significantly influence HIV vulnerability by shaping individuals’ risk behaviors, mental health, and engagement with prevention and care services [6]. SGM experience discrimination in healthcare, employment, housing, and public spaces, contributing to a cumulative burden of minority stress [7, 8]. Experiences of racism, homophobia, and transphobia intersect to uniquely and disproportionately affect SGM of color, leading to heightened psychosocial stress and increased engagement in behaviors that elevate HIV risk, such as condomless sex and substance use during sex [9, 10]. Discrimination has been shown to contribute to medical mistrust and to reduce access to HIV prevention and care services, including pre-exposure prophylaxis (PrEP) and routine HIV testing [11, 12]. Understanding how discrimination contributes to HIV acquisition and transmission disparities is especially important in the Southern United States, where over half of all new HIV diagnoses in the U.S. are reported [13]. A recent review found that perceived race-based discrimination in healthcare settings was associated with lower PrEP awareness among Black sexual and gender minority (SGM) individuals [14]. In contrast, a study in New York City found that sexual orientation discrimination—but not race-based discrimination—was associated with increased HIV transmission potential among Black and Latino SGM [15]. Experiences of race-based discrimination may differ in the Southern context. For example, among SGM in Tennessee, of whom 80% identified as non-Hispanic Black, discrimination, exposure to violence, and psychological distress were significantly interrelated [16]. In addition, several studies have shown that higher levels of discrimination may be a risk factor for recreational drug use, particularly among young Black Americans [17, 18]. Furthermore, other studies have demonstrated an association between increased sexual activity and recreational drug use among SGM. A qualitative study involving SGM and public health workers identified both stigma and social networks with certain risk factors—such as elevated substance use and sexual activity—as key barriers to reducing HIV transmission-acquisition risk and substance use behaviors in SGM communities [19–23].

Consequently, a better understanding of the relationship between discrimination, substance use, and HIV vulnerability among SGM persons of color in the Southern US needs further exploration. The goal of this analysis is to examine the relationship between everyday discrimination, HIV status, and having several sexual partners among a cohort of minority SGM in Raleigh-Durham, North Carolina.

## Methods

### Study Population

Data are from a cross-sectional study conducted among SGM of color in Raleigh-Durham, North Carolina, which asked participants about their sexual networks as well as experiences of discrimination. This quantitative study was designed to investigate networks with a potentially higher probability of HIV transmission-acquisition. Data were collected between June 16, 2022, and March 1, 2024. Eligibility criteria included age 18 years or older, self-identify as Black/African American or Latinx/Hispanic, assigned male sex at birth, able to provide consent in English, and report history of sex with another person assigned male sex at birth. In addition, people living with HIV must have met clinical eligibility criteria, which included those (1) newly diagnosed with HIV (prior 12 months), (2) HIV viral load > 200 copies/mL in the past 12 months, or (3) no viral load reported in the past 12 months and referred for bridge counseling services.

In-person recruitment occurred at UNC clinic, and we received passive referrals from the health department, community-based organizations, and local social venues over the study period. In addition, paid Meta and dating app advertisements, social media engagement, and a peer referral program were utilized. Approximately 51% were recruited from clinics, 29% from social media, and 20% from other locations. Persons interested in participating in the survey completed an online screening survey. A study coordinator screened participants to assess their eligibility and confirm their identity (i.e. via photo ID) prior to obtaining informed consent over telephone or secure Zoom. In total, there were 4,680 screeners, 4,192 of which were fraudulent (identified via IP address verification). Enrolled participants were then emailed their personal survey link, which remained active for 30 days. Participants were compensated with a $40 e-gift card upon survey completion. The number of enrolled participants was limited by ability to contact people who filled out the screening survey. The response rate was reflected by the number of enrolled participants who completed the survey divided by the number eligible. The screening survey and the social network survey were hosted on secure REDCap platforms [24, 25]. The study was approved by the Institutional Research Boards at the University of North Carolina at Chapel Hill and University of California at San Diego.

### Measures

The survey instrument took approximately 30 min to complete and covered questions about sociodemographics (i.e. age in continuous years, race, ethnicity, gender, sexual orientation (dichotomized as gay/bisexual/queer vs. other), employment (dichotomized as currently employed yes/no), education (dichotomized as completing some high school or less vs. greater than high school education), criminal justice involvement (ever spent time in jail/prison) number of places lived in the past 12 months, sexual partners, and an everyday discrimination scale ((EDS) described below). One socioeconomic variable we assessed was frequency of economic instability (e.g., difficult to pay rent/food/utilities) where participants answered often, sometimes, or never. We defined economic stability as those who answered often/sometimes vs. those who answered never.

Our outcome of interest was sexual activity, assessed as the number of sexual partners reported in the past 6-months. Participants were categorized into higher and lower sexual activity groups: high (> = 3 sexual partners) or low (0–2 sexual partners), as reporting three or more recent partners has been associated with incident infection in other high HIV prevalence populations [26].

For individual sexual risk assessment, participants answered questions about their STI history, HIV status, PrEP usage, and recent substance use (participants selected which licit and illicit drugs they used over the past 12-months from a list).

### Everyday Discrimination Scale

To measure everyday discrimination, participants answered the revised five-item EDS which has shown to “retain predictive validity consistent with the original scale” [27], and was selected due to its previous association with reduced HIV testing among Black populations in the U.S [28]. This scale asks about the frequency of daily experiences of discrimination and perceptions about the cause. Participants provided responses for how often they experienced a certain type of discrimination with a 6-point Likert scale, from “never” to “almost every day”, of the following five situations:

1. You are treated with less courtesy or respect than other people.
2. You receive poorer service than other people at restaurants or stores.
3. People act as if they think you are not smart.
4. People act as if they are afraid of you.
5. You are threatened or harassed.

These were followed by a question asking the participants what they believe are the main reasons for these experiences and were able to check all that apply, including gender, race, age, an aspect of physical appearance, sexual orientation, physical disability, and other (specify). We scored the EDS by a situation-based scoring system [27, 29], which sums the number of different situations respondents have experienced (0 = never, 1 = ever for each situation) for a score ranging from 0 to 5.

### Statistical Analysis

Multivariate logistic regression was used to assess the relationship between situation-based discrimination and frequent sexual activity (*≥* 3 partners vs. <3 partners in the past 6 months). Descriptive statistics included Wilcoxon rank sum test for non-normally distributed continuous variables, for normally distributed continuous variables we used Pearson’s correlation, and Fisher’s exact tests and Chi-squared tests were used for categorical variables. Variables significant at the *p* ≤ 0.1 level in bivariate logistic regression analyses were considered in the multivariable model. Initial models assessed age, race, ethnicity, gender, sexual orientation, employment, frequency of economic instability, housing, criminal justice involvement, recreational drug use, and HIV prevention and treatment history. The final model examining the relationship between EDS situation score (treated continuously) and sexual activity adjusts for age, ethnicity, frequency of economic instability, HIV status, and recreational drug use (dichotomized as any use past 12 months vs. not). All variables retained in the final model were significant at the *p* ≤ 0.05 level. Missing data were handled using listwise deletion. All analyses were conducted using R Statistical software v4.3.2 [30].

## Results

In total, 459 people were screened for the study and 235 met eligibility criteria. Of these 114 were enrolled and 100 completed the survey including the EDS instrument. Participants consisted of mostly Black/African American cisgender men who identified as gay, bisexual, or queer, with a small percentage of participants who identified as transgender or gender diverse (TGD) [Table 1]. STI history was prominent, with 65% having had an STI diagnosis in their lifetime and 55% living with HIV. Alcohol (66%), marijuana (57%), and recreational drug (37%) use were very high. The most common illicit recreation drugs were cocaine (14%) and methamphetamines (13%). Recreational drug use was significantly greater in those with more sexual partners (53% vs. 27%). While not significant, marijuana use tended to be greater in people with more partners (68% vs. 50%).

**Table 1:** Everyday discrimination is associated with higher sexual activity in SGM people of color in Raleigh-Durham, NC

		Sexual Activity Level				Unadjusted				Adjusted	
Characteristic	Overall	Low, *N* = 62^1^	High, *N* = 38^1^	N	OR_2_	95% CI^2^	p-value		OR_2_	95% CI^2^	p-value
EDS Situation Score	4 (2.00, 5)	3 (1.00, 5)	4 (3.00, 5)	100	1.49	1.15, 2.01	0.005*		1.76	1.25, 2.61	0.002*
Age	32 (26, 38)	33 (25, 39)	31 (27, 37)	97	0.99	0.95, 1.03	0.5		0.97	0.92, 1.02	0.2
Latino/Hispanic	21 (22%)	10 (17%)	11 (31%)	96	2.20	0.82, 5.97	0.12		1.92	0.54, 7.03	0.3
Black/African American	81 (81%)	53 (88%)	28 (74%)	99	1.39	0.60, 3.20	0.44				
Identifies as Cisgender Male	88 (90%)	53 (88%)	35 (92%)	98	1.54	0.40, 7.52	0.6				
Trans female	5 (5.1%)	3 (5.0%)	2 (5.3%)		–	–					
Other gender	5 (5.1%)	4 (6.7%)	1 (2.6%)		–	–					
Identify as Gay, Bisexual, or Queer	89 (89%)	57 (92%)	32 (84%)	100	0.47	0.13, 1.67	0.2				
High School or Less	25 (25%)	17 (27%)	8 (21%)	100	0.71	0.26, 1.80	0.5				
Often/Sometimes without money	60 (61%)	42 (69%)	18 (47%)	99	0.41	0.17, 0.93	0.035*		0.18	0.05, 0.54	0.004*
Ever in jail or prison	27 (27%)	18 (29%)	9 (24%)	99	0.79	0.30, 1.96	0.6				
Currently Employed	63 (64%)	36 (59%)	27 (73%)	98	1.88	0.79, 4.70	0.2				
Locations lived past 12 mo	2 (1, 2)	2 (1, 2)	1 (1, 2)	94	0.74	0.47, 1.13		0.2			
Diagnosed with HIV	55 (55%)	38 (61%)	17 (45%)	100	0.51	0.22, 1.15		0.11	1.60	0.49, 5.54	0.4
Currently on ART or PrEP	65 (65%)	37 (60%)	28 (74%)	100	1.89	0.80, 4.72		0.2			
Recreational Drug Use Past 12 months	37 (37%)	17 (27%)	20 (53%)	100	2.94	1.27, 6.97		0.013*	4.69	1.59, 15.5	0.007*
Alcohol	66 (66%)	38 (61%)	28 (74%)	99	1.93	0.80, 4.68		0.14			
Marijuana	57 (57%)	31 (50%)	26 (68%)	100	2.17	0.94, 5.17		0.07			

^1^Median (IQR); n (%). ^2^Wilcoxon rank sum test; Pearson’s Chi-squared test; Fisher’s exact test. *p-value < 0.05

**Fig. 1:**
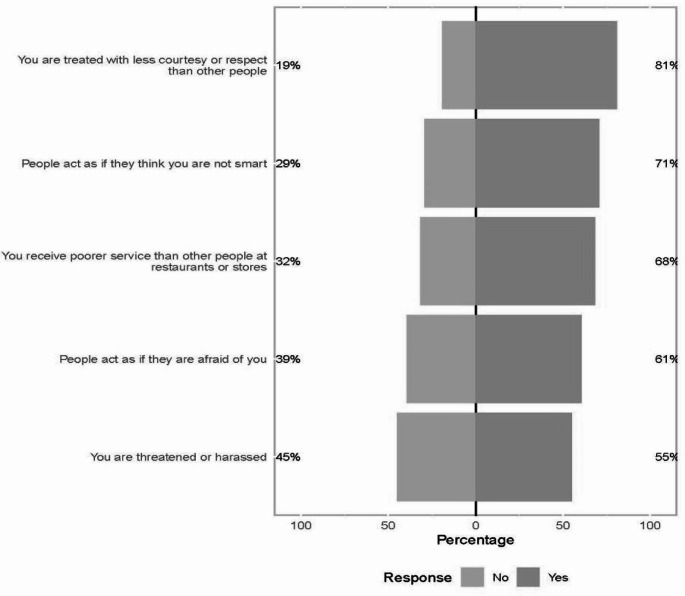
Prevalence of discrimination among SGM of color in Raleigh-Durham, NC. Response ‘no’ indicated in light gray. Response‘yes’ indicated in dark gray

In response to each question on the EDS, most participants reported experiencing every discriminatory situation; 81% had been treated with less courtesy or respect than other people, 71% experienced people act as if they are not smart, 68% received poorer service in restaurants or stores, 61% had people act as if they were afraid of them, and 55% were threatened or harassed (Fig. 1). The most frequently reported reasons for discrimination were race (56%), sexual orientation (48%), and some aspect of their physical appearance (39%). Interestingly, those with higher sexual activity reported an aspect of their physical appearance being a reason they experienced discrimination significantly more than those with lower sexual activity reported an aspect of their physical appearance being a reason they experienced discrimination (54% vs. 28%, *p* = 0.01). Overall, EDS Situation Scores were high with a median score of 4 [IQR 2, 5]. EDS situation scores were significantly higher among those with high sexual activity (*≥* 3 partners in the past 6-months) compared with < 3 partners (*p* = 0.007), and did not differ by race/ethnicity (*p* = 0.83).

In multivariate analysis, greater EDS situation score was associated with greater odds of high sexual activity (Table 1) (adjusted OR 1.76 [95% CI 1.25, 2.61] *p* = 0.002). Recent recreational drug use was also highly associated with higher sexual activity (adjusted OR 4.69 [95% CI 1.59, 15.5] *p* = 0.007). Interestingly, economic instability was associated with lower sexual activity (adjusted OR 0.18 [95% CI 0.05, 0.54] *p* = 0.004).

## Discussion

This study is among the first in the Southern United States to examine the relationship between increased sexual activity and experiences of discrimination among sexual and gender minority (SGM) persons of color. Alarmingly, our cohort reported high rates of everyday discrimination, with over half reporting exposure to all measured situations and 80% reporting at least one. In Southern U.S. contexts, everyday discrimination and HIV-related stigma frequently intersect, compounding healthcare avoidance and undermining engagement with HIV prevention and treatment services. For example, individuals living with HIV in the South often report high levels of experienced, perceived, and internalized stigma, all of which negatively affect care engagement [31]. A study of women living with HIV in North Carolina found that both everyday discrimination and group-based medical mistrust were linked to depressive symptoms, which impaired adherence to treatment and clinic attendance [32].

The association between daily discrimination and increased sexual activity observed in our sample may contribute to heightened vulnerability to HIV transmission and acquisition among Southern SGM persons of color. Qualitative studies indicate that discrimination is linked to psychological distress and heightened perceptions of HIV vulnerability, particularly in young Black SGM populations [33]. Similar findings have emerged in other regions. In California, a majority of young Black SGM reported frequent experiences of discrimination [34], particularly in healthcare settings, which may limit engagement with HIV prevention and treatment services. A study in Vancouver found that Black LGBTQ + Canadians who experienced racism also faced difficulty accessing healthcare and were less likely to receive HIV testing [35]. Similarly, U.S.-based research has shown that institutional discrimination often leads LGBTQ + individuals—especially racial and ethnic minorities—to avoid healthcare altogether [36]. Given the high levels of everyday discrimination reported in our Southern cohort—currently residing in the epicenter of the U.S. HIV epidemic—future prevention research should explore how racism and institutional discrimination influence health-seeking behaviors and beliefs that affect HIV vulnerability.

We also found a significant association between recent recreational drug use and increased sexual activity. Interestingly, economic instability was linked to lower sexual activity—contrasting with prior literature that highlights relationships among economic hardship, discrimination, and transactional sex. For example, studies of transgender and gender diverse (TGD) individuals have demonstrated that racism and transphobia contribute to increased substance use and transactional sex among young TGD people of color [37]. The low representation of TGD individuals in our sample may explain this divergence. In our context, economic stability may facilitate access to recreational drugs, whereas financial insecurity might limit both drug use and sexual activity. Moreover, chronic stress associated with economic hardship and discrimination could reduce sexual desire, offering another explanation for this inverse relationship [38].

In addition to everyday discrimination, HIV-related stigma significantly influences engagement with prevention services, including pre-exposure prophylaxis (PrEP). Studies have documented that discrimination in healthcare settings based on race and sexual orientation is associated with lower awareness and uptake of PrEP among Black SGM [13]. Moreover, the intersection of multiple stigmatized identities—related to race, sexual orientation, gender, and HIV status—may compound barriers to PrEP and antiretroviral therapy use. Together with our findings that increased discrimination is linked to behaviors associated with HIV risk, these data underscore the need to understand the interplay of multiple forms of stigma. Future research should further investigate the relationship between discrimination and PrEP use among SGM people of color to inform targeted, effective HIV prevention strategies.

## Limitations

Our study is limited by cross-sectional design which inherently limits inference of causality. Participants were largely recruited from convenience sampling and may have potential self-selection participation bias. Convenience sampling reduces the generalizability of the sample as non-random selection may result in certain groups being over or underrepresented. The small sample size limited the number of variables we could include in the regression model without risking overfitting the model. However, six covariates is an appropriate number for our sample size [39]. Additionally, the study was conducted solely in Raleigh-Durham, North Carolina, which may not reflect the experiences of SGM of color in other areas. This limited geographic scope constrains broader applicability of the results. However, Raleigh-Durham is a rapidly growing urban area in the U.S. South, a region disproportionately affected by HIV and historically underrepresented in HIV research. As such, the study provides important insights into the intersection of discrimination, sexual behavior, and substance use among SGM of color in a high-priority setting for HIV prevention. The reliance on self-reported measures introduces the potential for recall and social desirability biases, particularly around sensitive topics like substance use and sexual behavior. Although efforts were made to recruit TGD participants, most respondents were cisgender men, limiting insights into the experiences of TGD individuals. Finally, while we assessed everyday discrimination broadly, we could not fully disentangle the complex intersections of racism, homophobia, and HIV-related stigma, which may have differential impacts on HIV vulnerability. However, we accounted for sampling bias by using a wide variety of recruitment methods, both in person and online. All enrolled participants were verified as eligible and consented following identification, thus curtailing fraudulent participants, which is typically a challenge with online surveys.
